# Supplementary material for: 3D architecture and complex behavior along the simple central San Andreas fault
Source: Nat Commun. 2024 Jun 25;15:5390. doi: 10.1038/s41467-024-49454-z (PMC11199709; doi:10.1038/s41467-024-49454-z)
Supplement: Supplementary file 1 — Supplementary Information [file 41467_2024_49454_MOESM1_ESM.pdf]

Supplementary Information for

3D architecture and complex behavior along the simple central San Andreas fault

Yifang Cheng *et al.*

*Corresponding author. Email: chengyifang@tongji.edu.cn

This PDF file includes:

Figs. 1 to 18

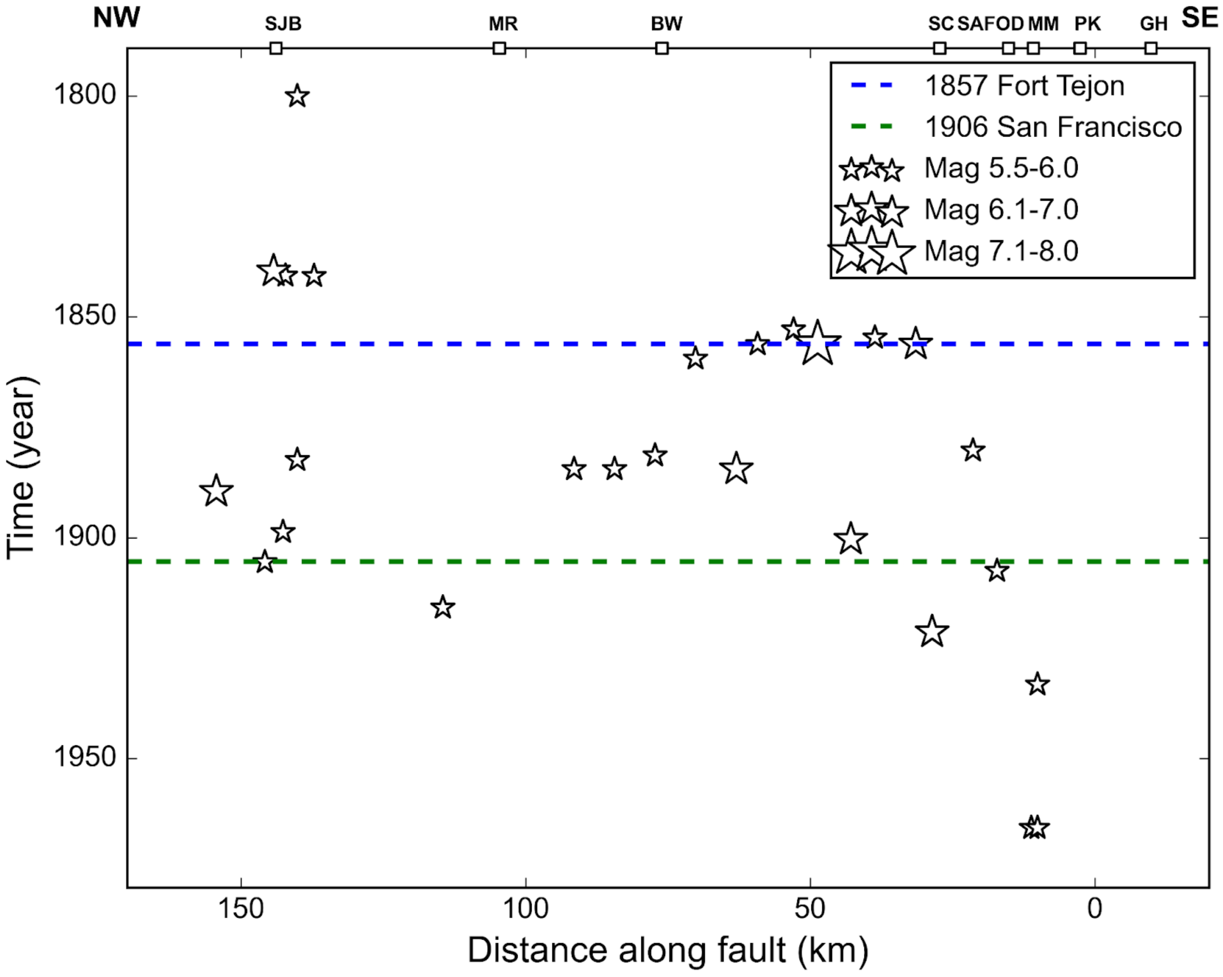

Supplementary Fig. 1. Spatiotemporal distribution of historical $M_w \geq 5.5$ earthquakes before 1984 presumed to have occurred on the CSAF from Toppozada et al., (2002).

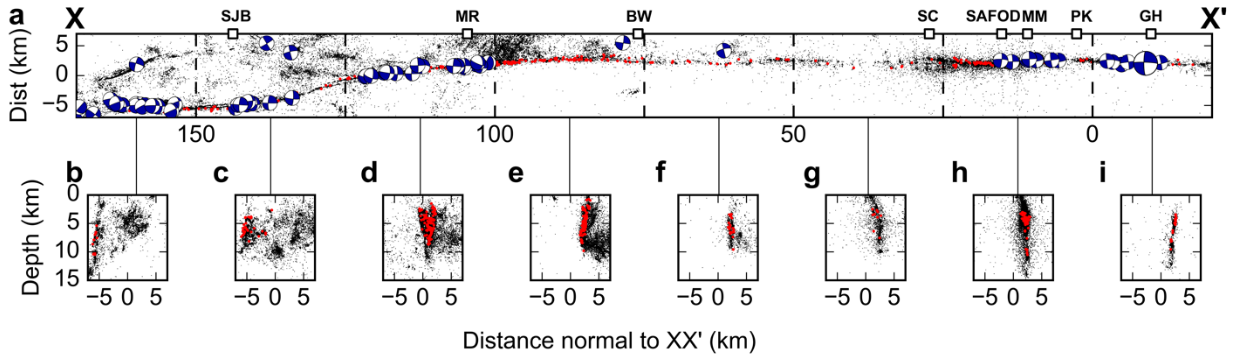

Supplementary Fig. 2. a Rotated map view, **b-i** cross-section views of seismicity (black dots), M4.0 earthquakes (blue beachballs), and repeating earthquakes (Waldhauser and Schaff, 2021; red dots) along the Central San Andreas Fault. The local coordinate system has its origin at latitude 35.867°N, longitude 120.447°W and is oriented N42°W. SJB: San Juan Bautista, MR: Melendy Ranch; BW: Bitterwater; SC: Slack Canyon; SAFOD: San Andreas Fault Observatory at Depth; MM: Middle Mountain; PK: Parkfield; GH: Gold Hill.

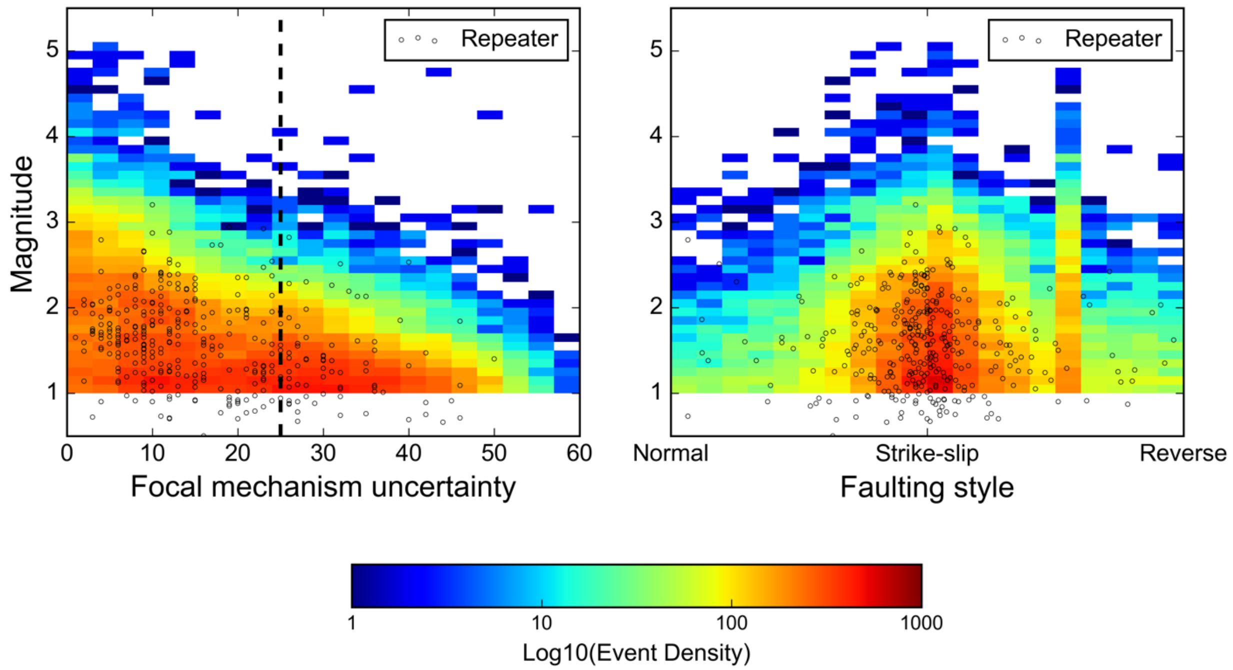

Supplementary Fig. 3. a 2D histogram of focal mechanism uncertainty versus magnitude of $M \geq 1.0$ earthquake located within 1-km epicentral distance from the major fault. **b** faulting style versus magnitude of $M \geq 1.0$ earthquakes located within 1-km epicentral distance from the major fault with focal mechanism uncertainty less than 25 degrees. Black circle denotes focal mechanism of repeating earthquake sequence.

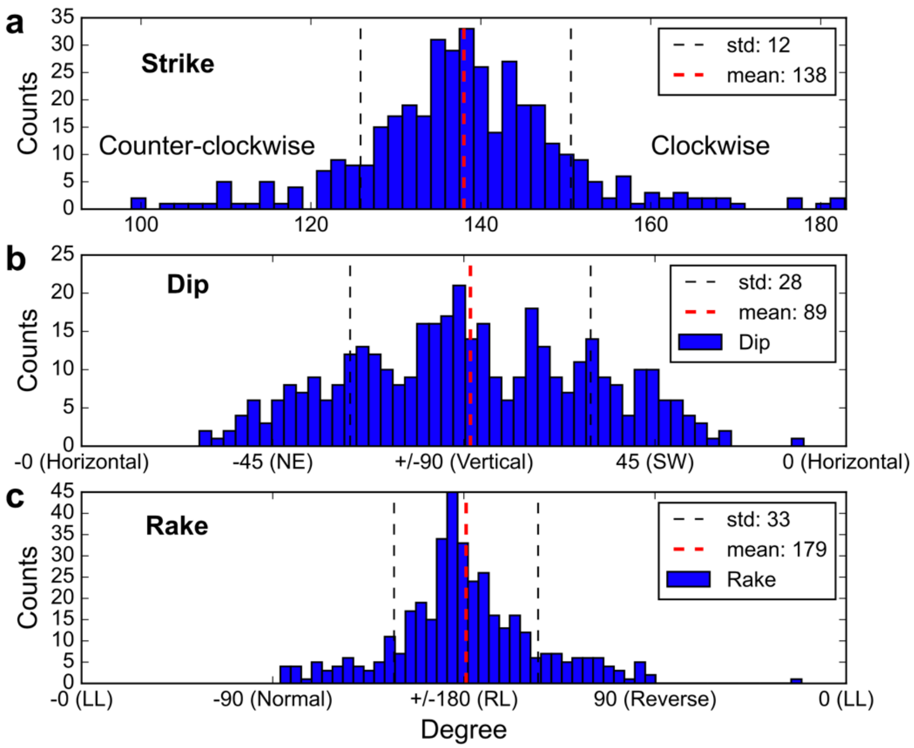

Supplementary Fig. 4. Histogram of the **a** strike, **b** dip, and **c** rake of the preferred nodal plane of the repeating earthquake sequences with smaller azimuthal difference from the main-fault, N42°W-striking orientation. Red and black dashed vertical lines denote the mean and one standard deviation, respectively.

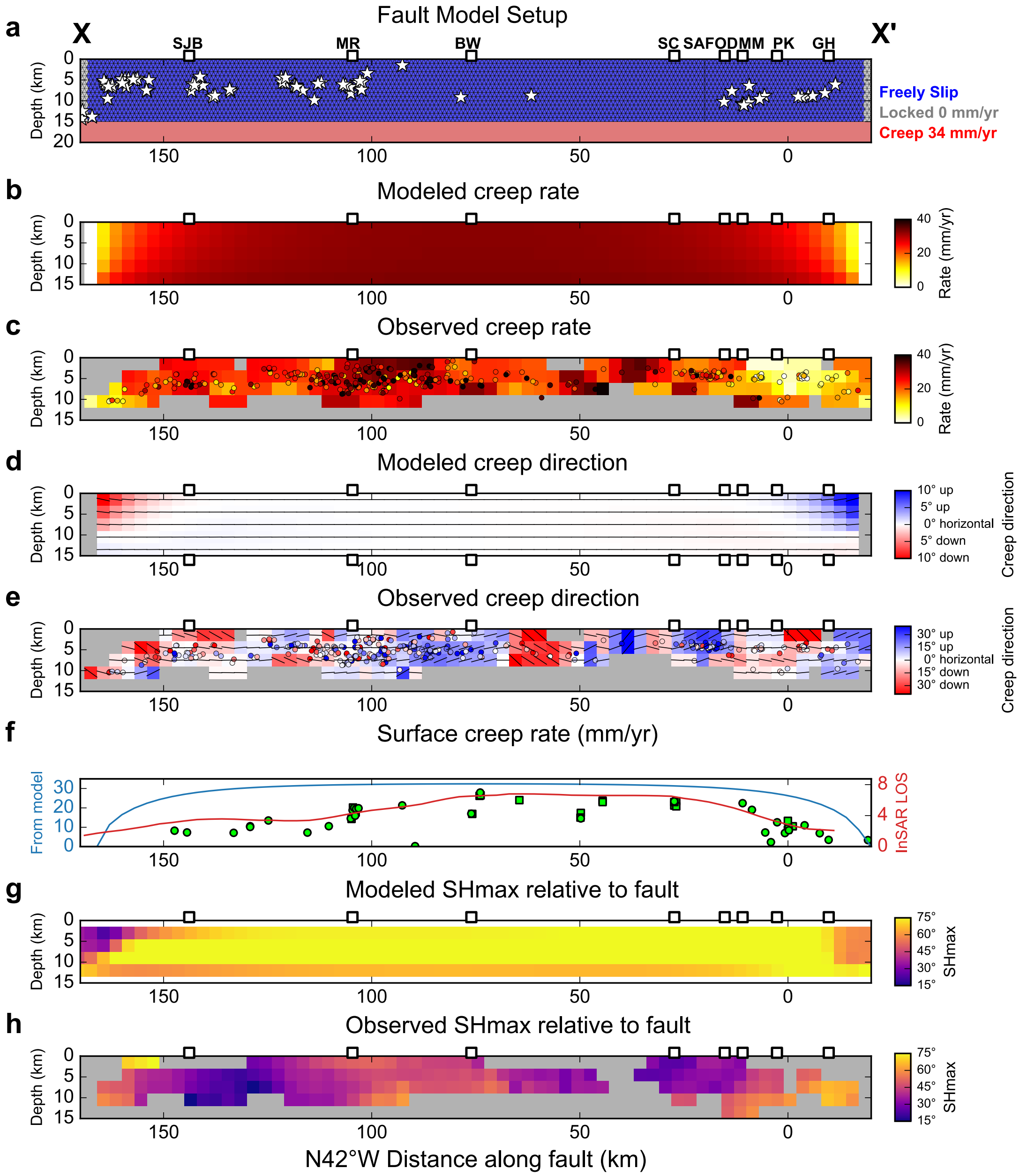

Supplementary Fig. 5. a Fault model B setup of a fully uncoupled CSAF with freely slipping zones (blue), locked sections (gray) and steadily creeping zones (red). The shallow fault is fully coupled beyond 166 km NW and -17km SE of the fault. The size of each fault patch is 3×3km. White stars denote $M_l \geq 4.0$ earthquakes. **b** Modeled and **c** observed fault creep rate estimated from the occurrence of repeating earthquakes. **d** Modeled and **e** observed fault creep direction estimated from the rake and dip of repeating earthquake focal mechanisms. Black thin line denotes local creep direction on the NE side of the fault. **f** Modeled (blue) and observed surface creep rate (red) estimated from InSAR data (Jolivet et al., 2014), creepmeters (green circles) and alignment arrays (green squares) (Titus et al., 2006). **g** The angle between the main fault and the modeled off-fault maximum horizontal stress orientation (θ) (from the area 1.5km NE of the main fault). **h** The angle between the main fault and the observed maximum horizontal stress orientation calculated from M1.0 focal mechanisms located within 2km around the fault trace.

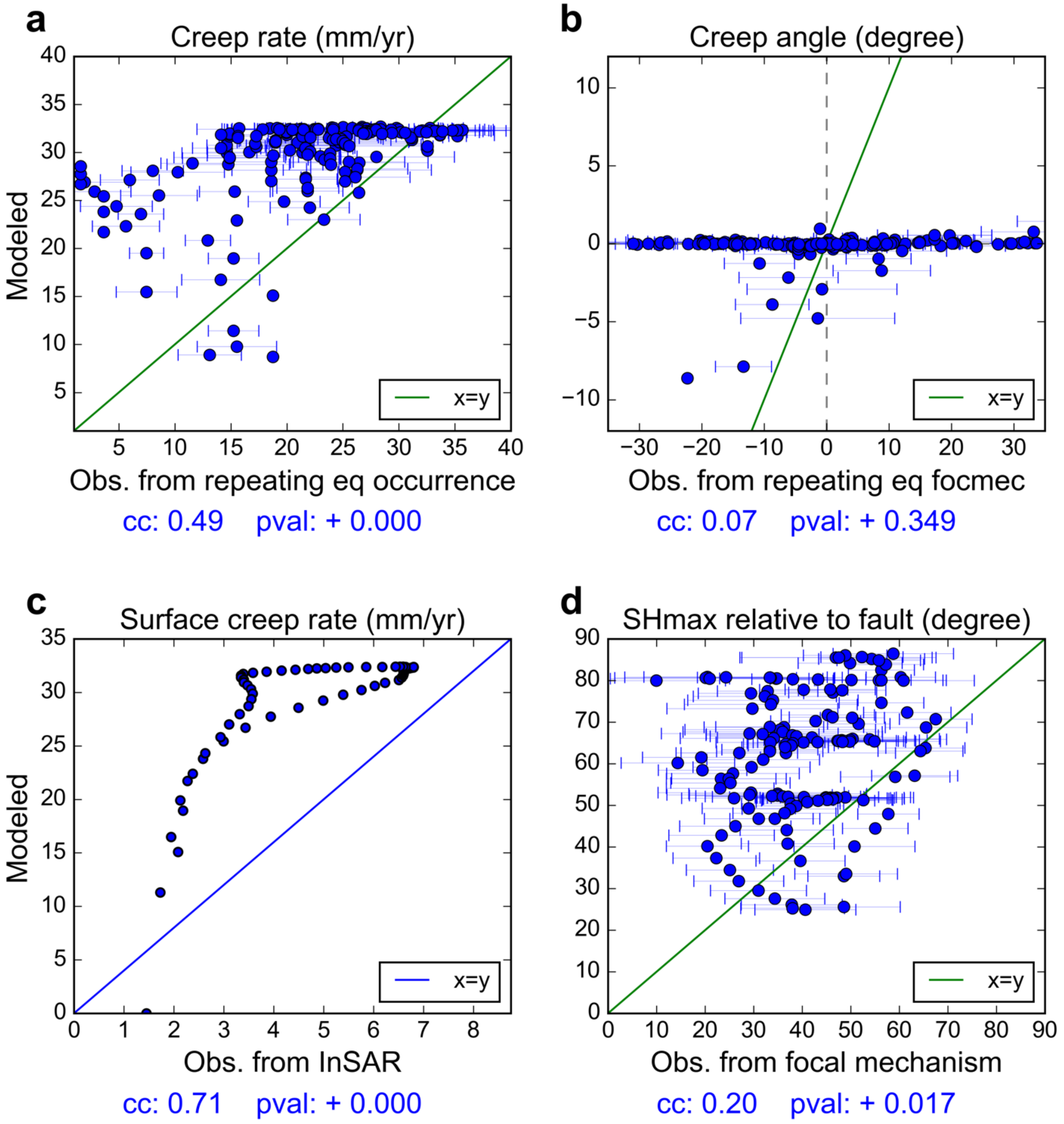

Supplementary Fig. 6. Point-to-point comparisons of the **a** slip rate, **b** slip direction, **c** surface creep rate, and **d** the angle between main fault and the off-fault maximum horizontal stress orientation θ (from the area 1.5km NE to the main fault) modeled from the fault coupling model B (Y-axes) and those estimated from **a** repeating earthquake occurrence rates, **b** repeating earthquake focal mechanisms, **c** InSAR data, and **d** M1.0 focal mechanisms (X-axes). (See Fig. 9 for the data used for comparison)

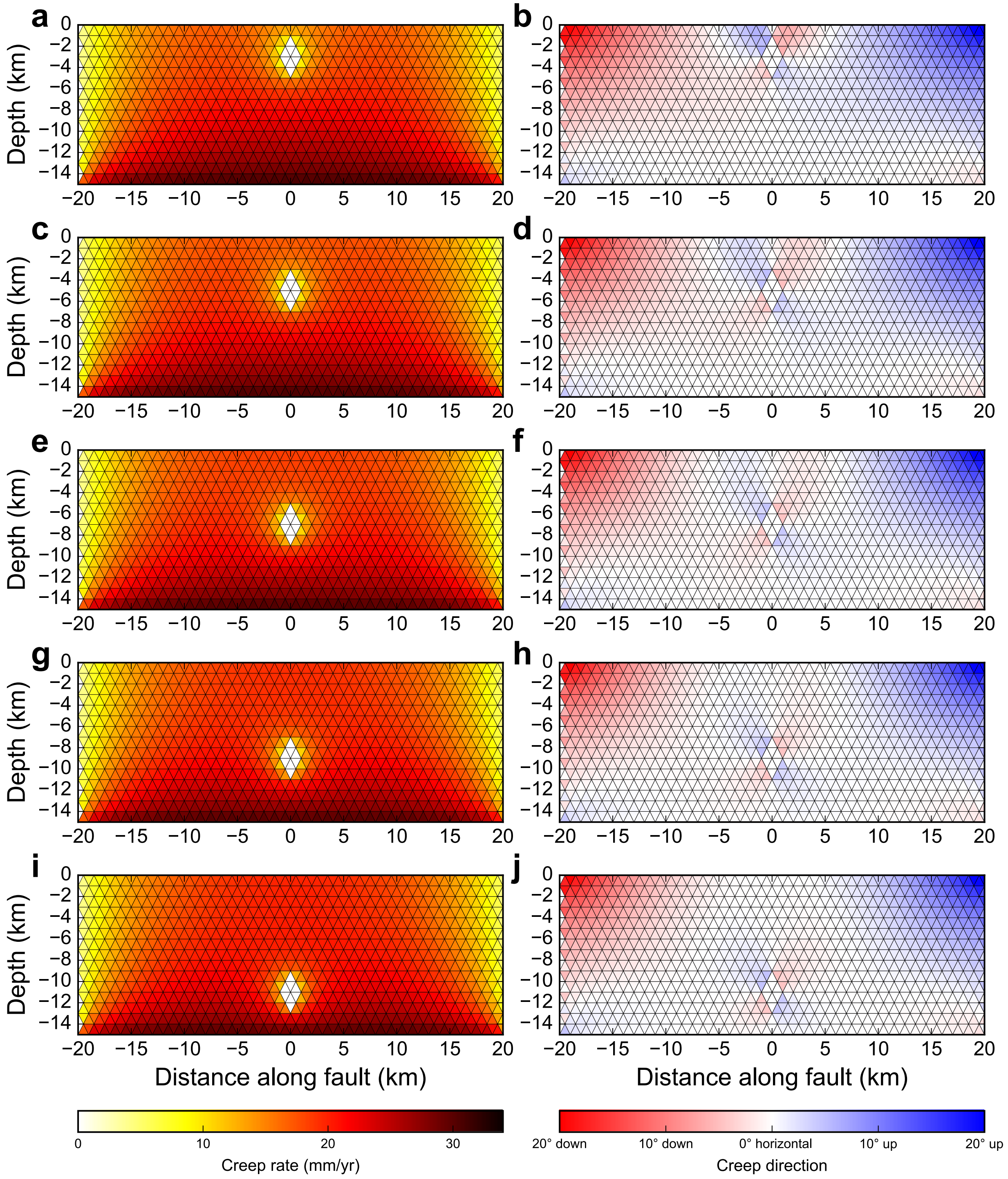

Supplementary Fig. 7. Modeled fault creep rate (left side) and creep direction (right side) along a section of a creeping fault, which is flanked by locked segments at both ends and contains a locked patch in the middle. Each row presents the results with the locked patch at varying depths.

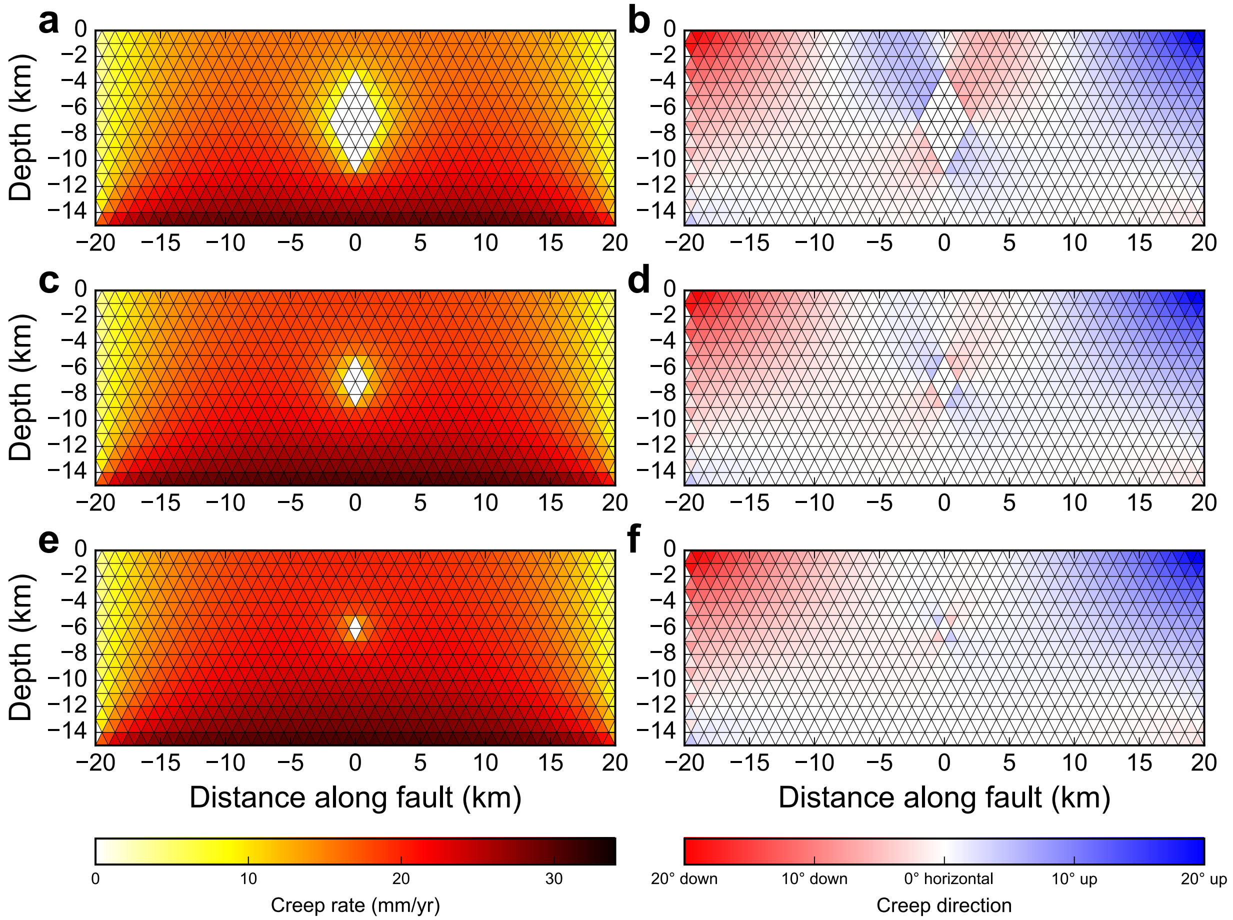

Supplementary Fig. 8. Modeled fault creep rate (left side) and creep direction (right side) along a section of a creeping fault, which is flanked by locked segments at both ends and contains a locked patch in the middle. Each row presents the results for varying sizes of the locked patch.

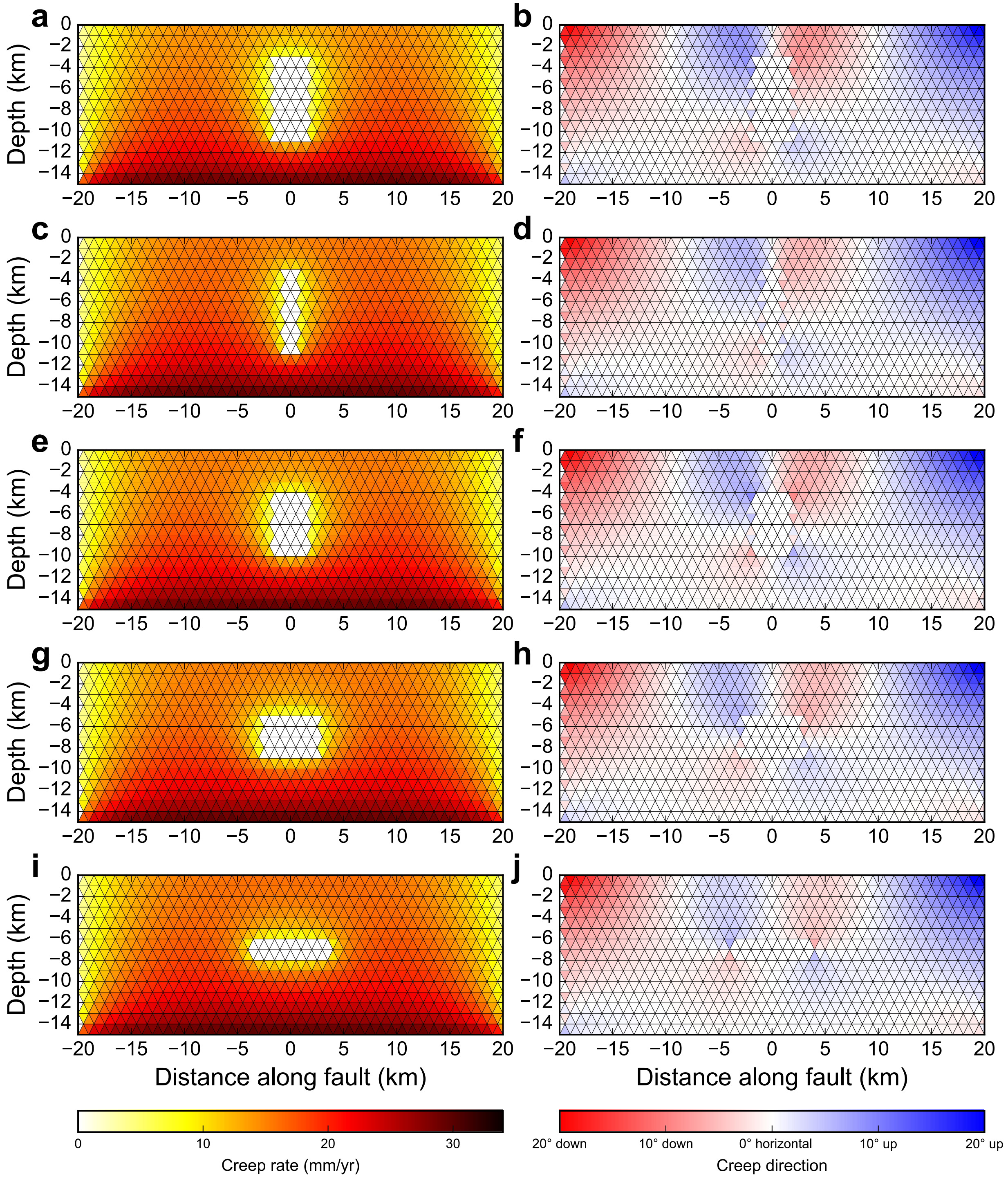

Supplementary Fig. 9. Modeled fault creep rate (left side) and creep direction (right side) along a section of a creeping fault, which is flanked by locked segments at both ends and contains a locked patch in the middle. Each row presents the results for varying shapes of the locked patch.

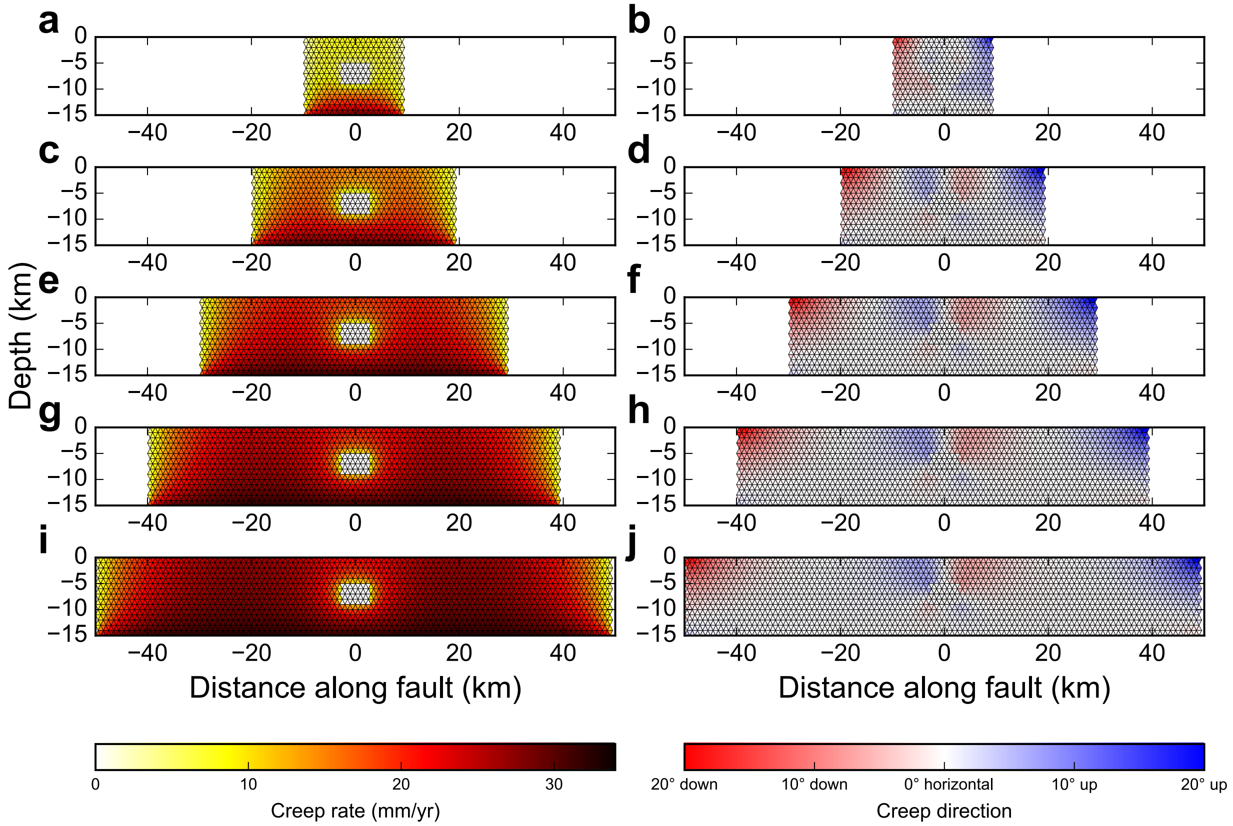

Supplementary Fig. 10. Modeled fault creep rate (left side) and creep direction (right side) along a section of a creeping fault, which is flanked by locked segments at both ends and contains a locked patch in the middle. Each row presents the results for varying lengths of the creeping fault segment.

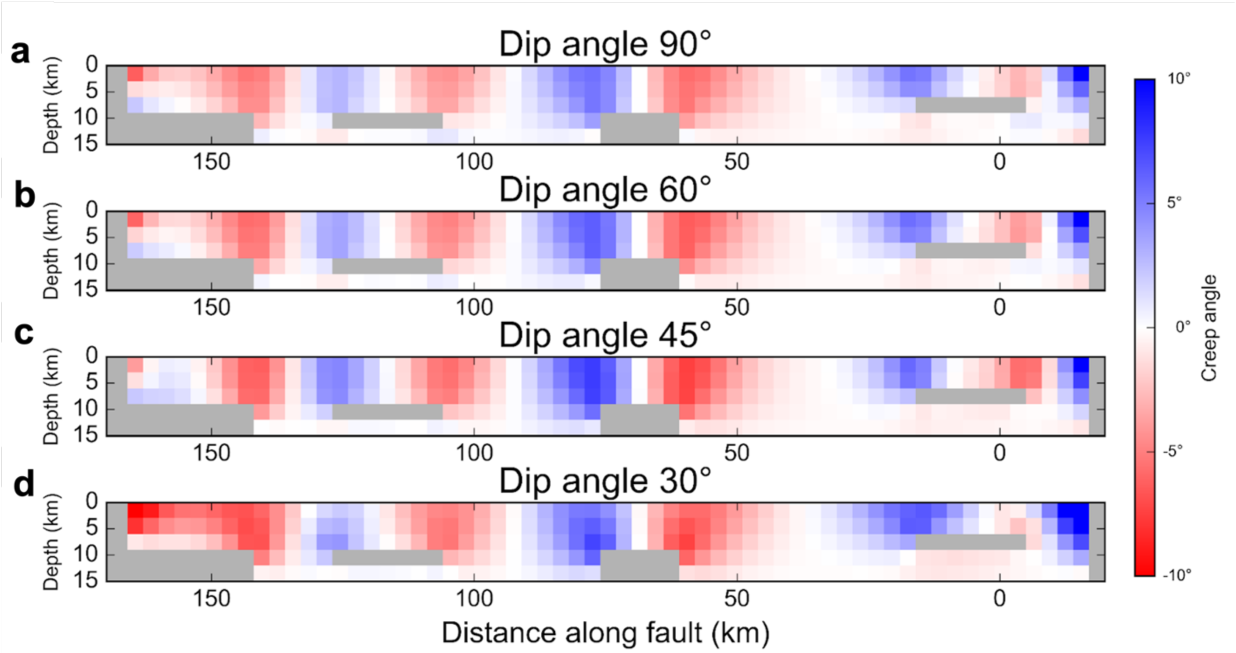

Supplementary Fig. 11. Modeled fault creep direction using fault model A with different, but uniform, SW dip angles. Positive creep directions indicate a NE-side-up dip-slip component.

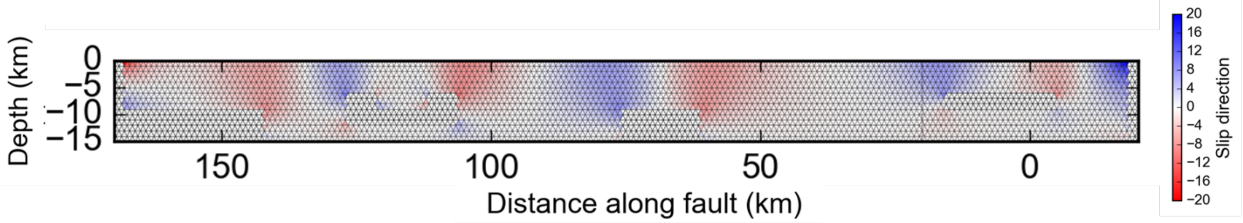

Supplementary Fig. 12. Fine-scale distribution of modeled fault creep direction for fault model A.

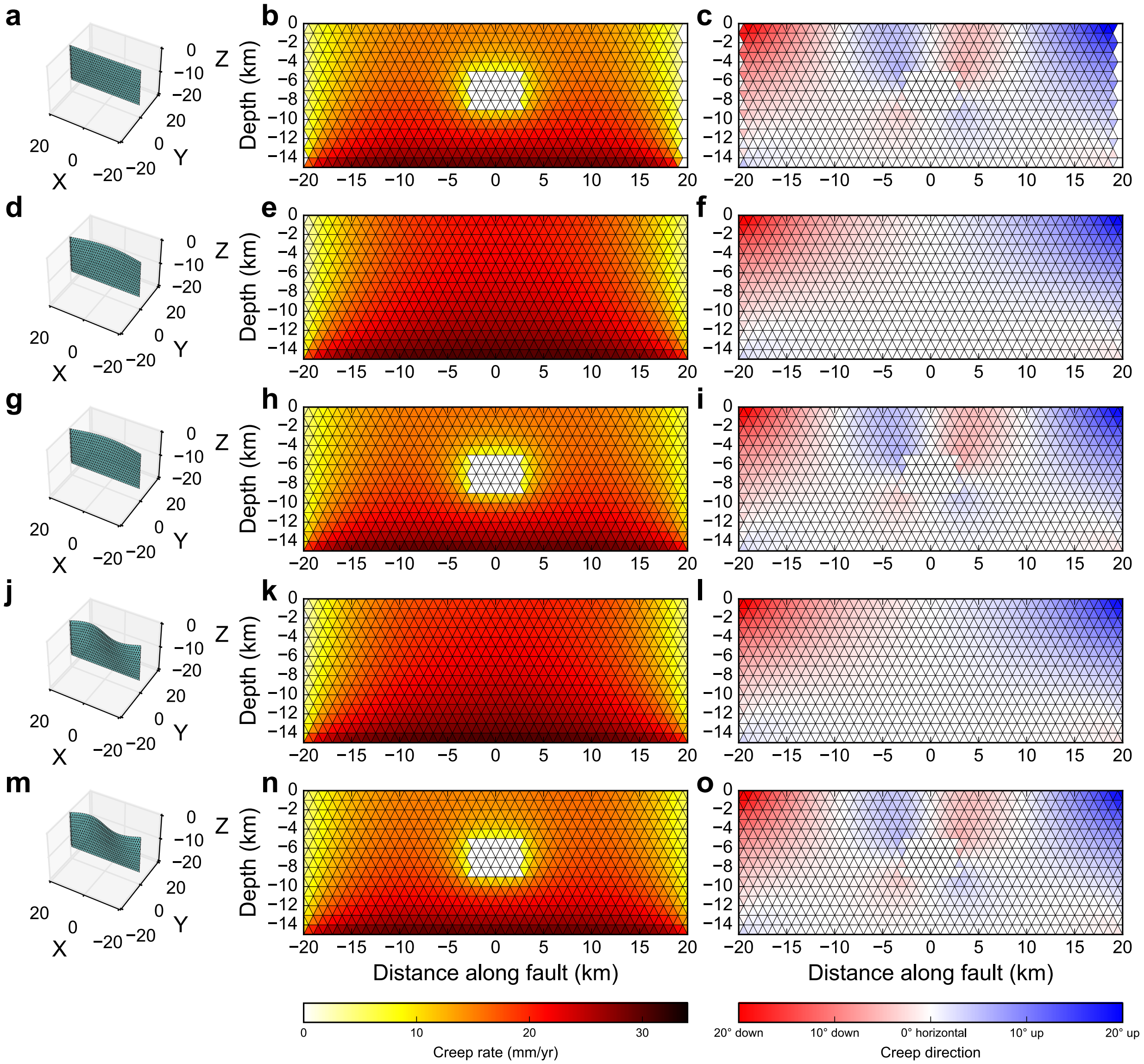

Supplementary Fig. 13. Model fault creep rate along X direction x (2nd column) and creep direction (3rd column) along a section of a creeping fault with different geometry (1st column), which is flanked by locked segments at both ends. Note that the creep direction angle here is not the creep angle on the fault plane but is determined from the ratio between the creep rate along X direction x and that along Z direction z ($\arctan\left(\frac{x}{z}\right)$; 3rd column). Each row shows the results for the creeping fault, detailing scenarios with and without a locked patch in the middle.

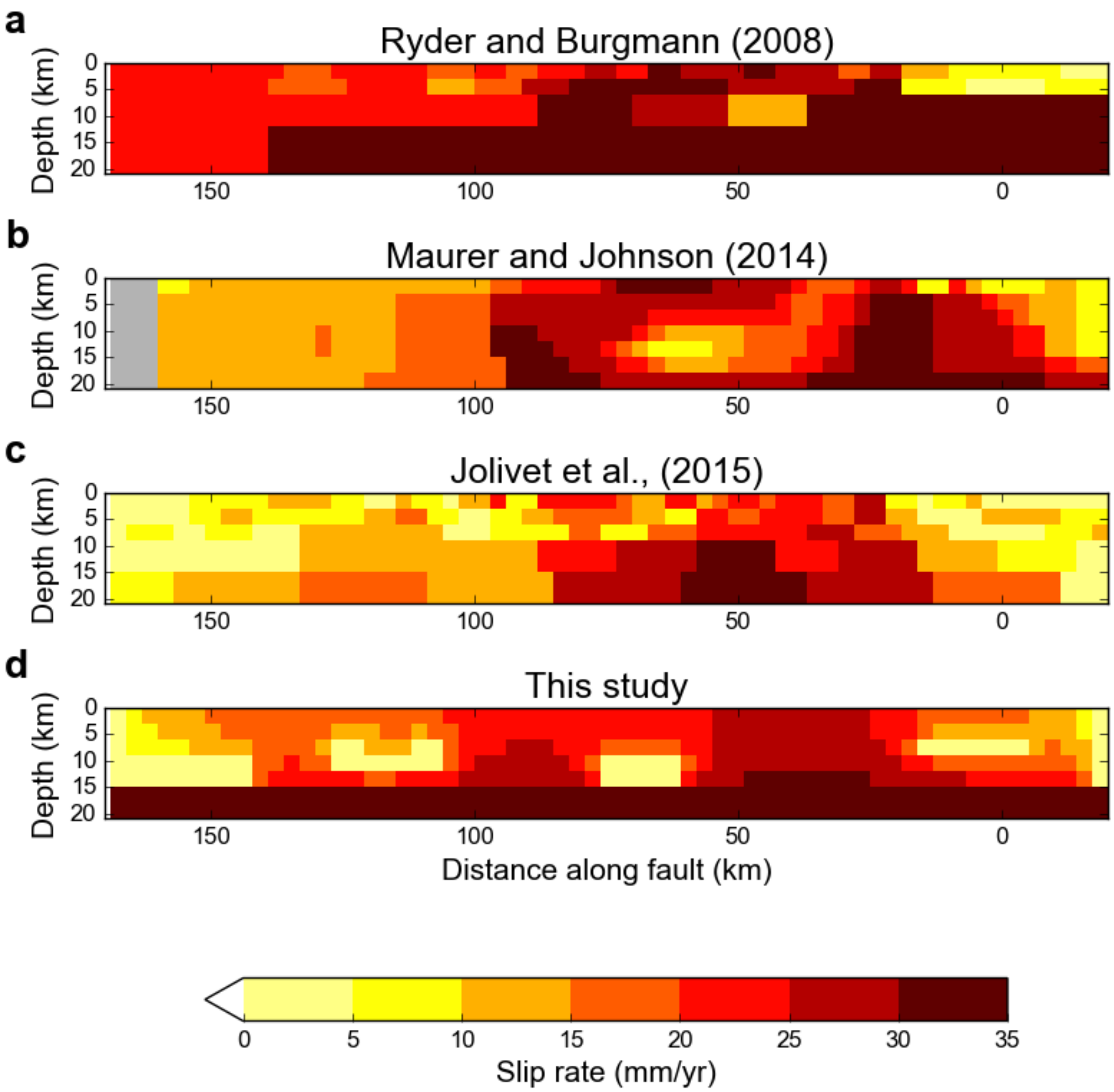

Supplementary Fig. 14. Comparison of several fault coupling models on the central San Andreas Fault. Collection of the modeled distributions of subsurface inter-seismic fault slip rate on the CSAF from **a** Ryder and Bürgmann (2000), **b** Maurer and Johnson (2014), **c** Jolivet et al., (2015), and **d** this study. All models shown at the same spatial and color sale.

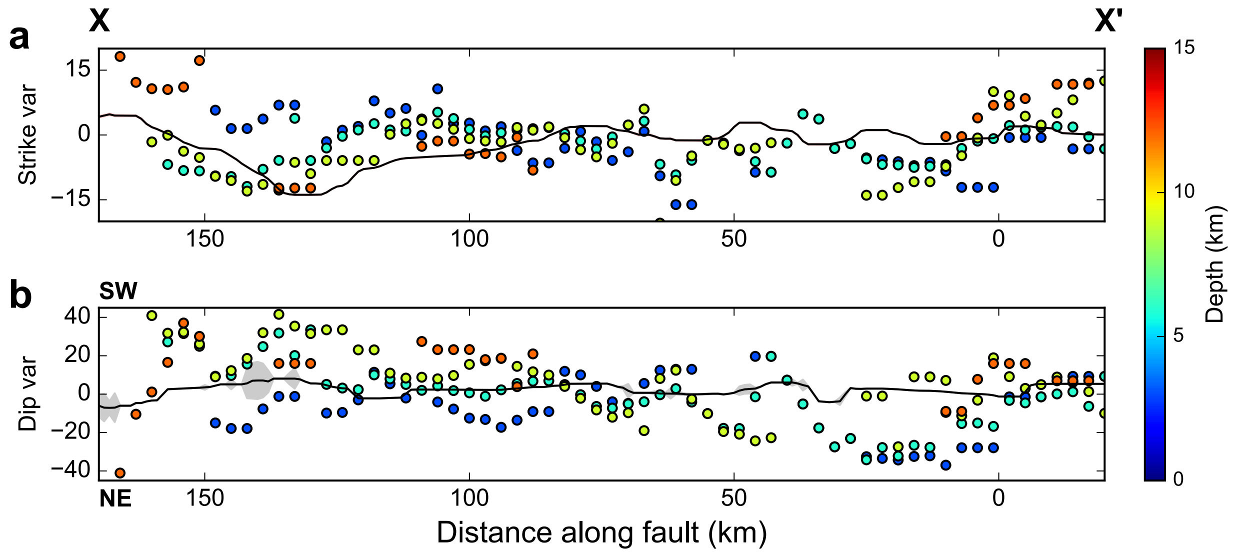

Supplementary Fig. 15. Comparisons of main fault **a** strike and **b** dip angles estimated from seismicity (Fig 2; black line) and the focal mechanisms of repeating earthquake sequences (fig. S4, B and C; colored dots). The gray areas denote the uncertainty of the estimated strike and dip orientation from seismicity. The focal mechanisms of repeating earthquake sequences have uncertainties (one standard deviation of a set of acceptable focal mechanisms considering the actual focal mechanism variations in each sequence and the expected errors in input data) less than 25 degrees.

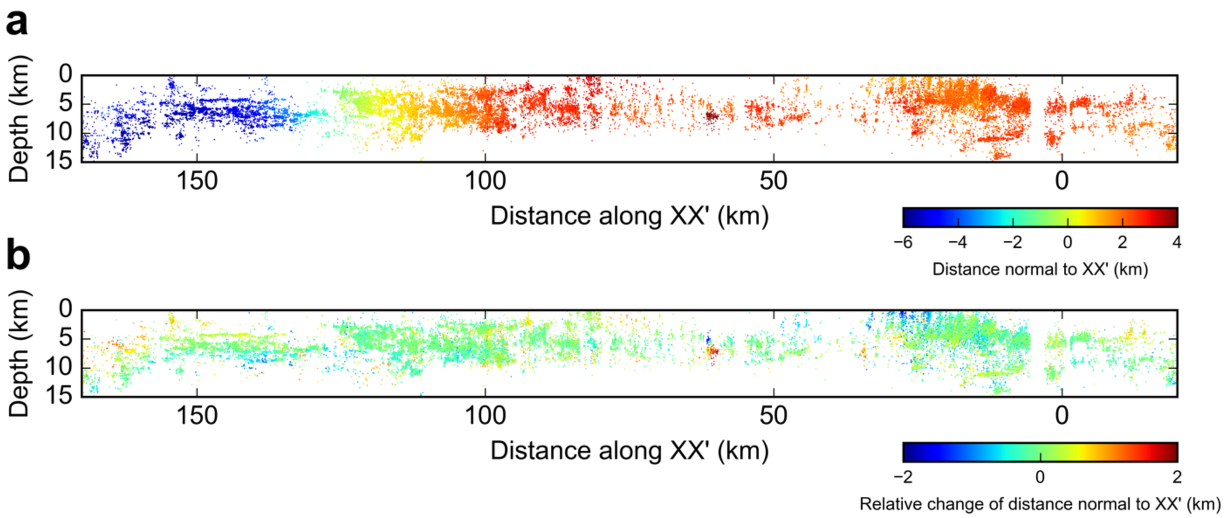

Supplementary Fig. 16. Cross-section view of M≥ 1.0 earthquakes within 1-km epicentral distance from the horizontal trace determine from repeating earthquakes. Each earthquake is colored by the **a** distance normal to XX' and **b** relative change of distance normal to XX' along depth.

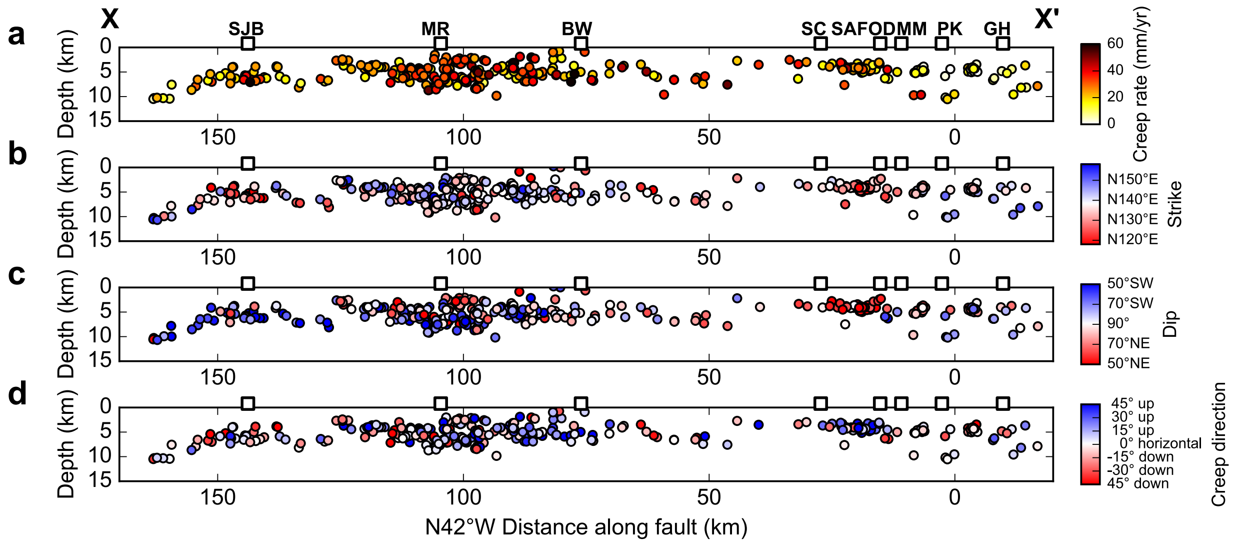

Supplementary Fig. 17. Cross-section view of repeating earthquake sequences colored by **a** creep rate estimated from occurrence rate, **b** preferred strike angle, **c** dip angle, and **d** creep direction estimated from their focal mechanism. The data in **a** and **d** are used to estimate the creeping rate and direction in each fault patch in Fig 4**c** and 4**e** in the main text, respectively. Positive creep directions indicate a NE-side-up dip-slip component.

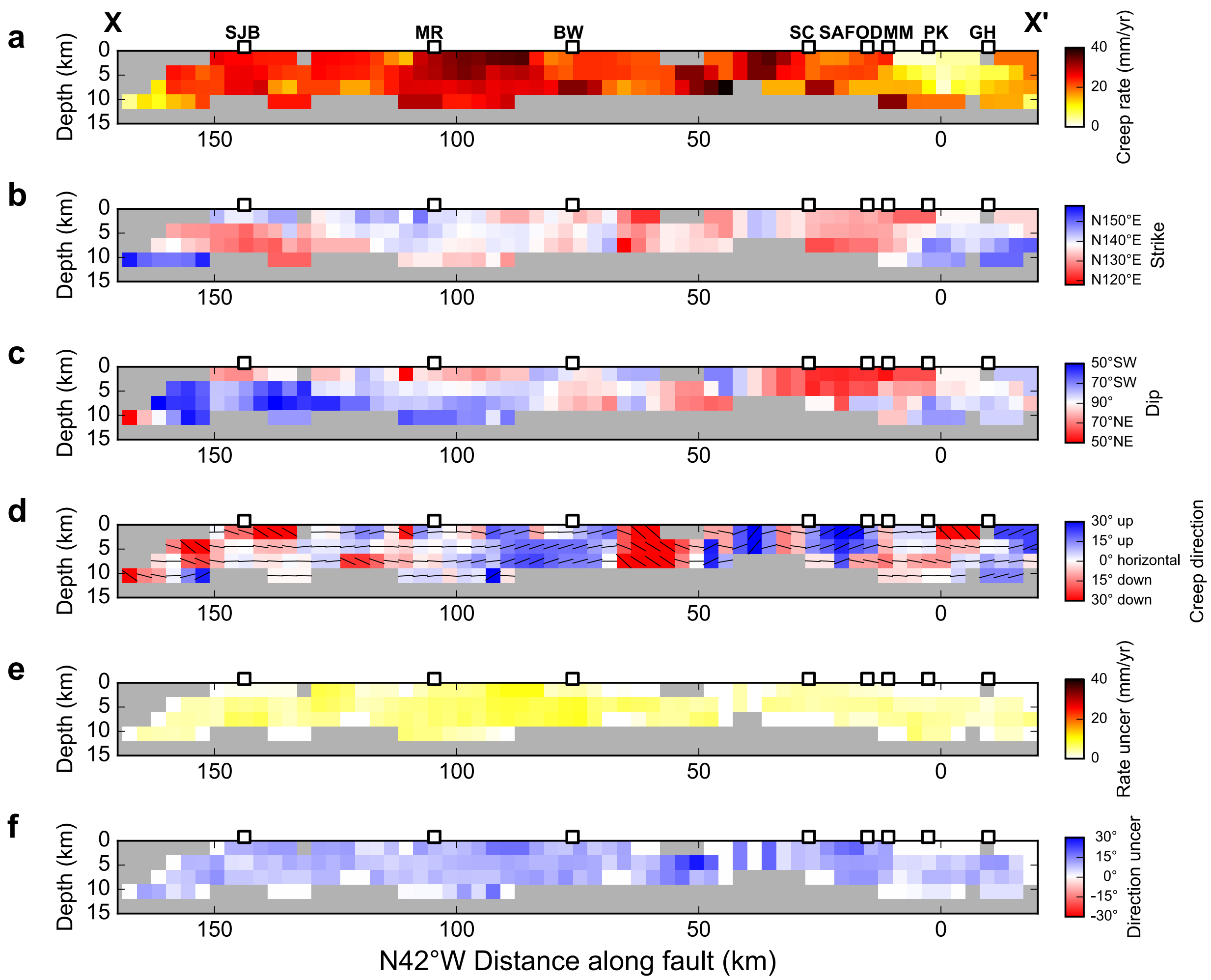

Supplementary Fig. 18. Spatial variation of the **a** fault creep rate, **b** strike, **c** dip, **d** creep direction, **e** creep rate uncertainty and **f** creep direction uncertainty of NE side of the fault estimated from repeating earthquakes on each fault patch (See Fig. 4 in the main text). The size of each fault patch is 3×3 km. Thin short lines in **d** denote creep direction of each fault patch.
