# Supplementary material for: 3D architecture and complex behavior along the simple central San Andreas fault
Source: Nat Commun. 2024 Jun 25;15:5390. doi: 10.1038/s41467-024-49454-z (PMC11199709; doi:10.1038/s41467-024-49454-z)
Supplement: Supplementary file 3 — Reporting Summary [file 41467_2024_49454_MOESM3_ESM.pdf]

nature portfolio

Corresponding author(s): Yifang Cheng

Last updated by author(s): May 13, 2024

Reporting Summary

Nature Portfolio wishes to improve the reproducibility of the work that we publish. This form provides structure for consistency and transparency in reporting. For further information on Nature Portfolio policies, see our Editorial Policies and the Editorial Policy Checklist.

Statistics

For all statistical analyses, confirm that the following items are present in the figure legend, table legend, main text, or Methods section.

n/a	Confirmed	
☐	☒	The exact sample size (*n*) for each experimental group/condition, given as a discrete number and unit of measurement
☐	☒	A statement on whether measurements were taken from distinct samples or whether the same sample was measured repeatedly
☒	☐	The statistical test(s) used AND whether they are one- or two-sided<br>*Only common tests should be described solely by name; describe more complex techniques in the Methods section.*
☐	☒	A description of all covariates tested
☐	☒	A description of any assumptions or corrections, such as tests of normality and adjustment for multiple comparisons
☐	☒	A full description of the statistical parameters including central tendency (e.g. means) or other basic estimates (e.g. regression coefficient) AND variation (e.g. standard deviation) or associated estimates of uncertainty (e.g. confidence intervals)
☒	☐	For null hypothesis testing, the test statistic (e.g. *F*, *t*, *r*) with confidence intervals, effect sizes, degrees of freedom and *P* value noted<br>*Give P values as exact values whenever suitable.*
☒	☐	For Bayesian analysis, information on the choice of priors and Markov chain Monte Carlo settings
☒	☐	For hierarchical and complex designs, identification of the appropriate level for tests and full reporting of outcomes
☐	☒	Estimates of effect sizes (e.g. Cohen's *d*, Pearson's *r*), indicating how they were calculated

Our web collection on statistics for biologists contains articles on many of the points above.

Software and code

Policy information about availability of computer code

Data collection	None
Data analysis	Python 3.9

For manuscripts utilizing custom algorithms or software that are central to the research but not yet described in published literature, software must be made available to editors and reviewers. We strongly encourage code deposition in a community repository (e.g. GitHub). See the Nature Portfolio guidelines for submitting code & software for further information.

Data

Policy information about availability of data

All manuscripts must include a data availability statement. This statement should provide the following information, where applicable:

- Accession codes, unique identifiers, or web links for publicly available datasets
- A description of any restrictions on data availability
- For clinical datasets or third party data, please ensure that the statement adheres to our policy

The InSAR surface slip-rate estimates were obtained from Jolivet et al., (2015)10. The repeating earthquake catalog was obtained from Waldhauser and Schaff (2021)28. The relocated earthquake catalog was obtained from Waldhauser and Schaff (2008)24. The earthquake phase information and seismic waveforms are taken from the Northern California Earthquake Data Center, Northern California Seismic Network (https://ncedc.org/ncsn/). The estimated earthquake focal mechanisms are available at Mendeley Data (https://data.mendeley.com/datasets/34szj3jm6k/1).

Research involving human participants, their data, or biological material

Policy information about studies with human participants or human data. See also policy information about sex, gender (identity/presentation), and sexual orientation and race, ethnicity and racism.

Reporting on sex and gender	Not applicable
Reporting on race, ethnicity, or other socially relevant groupings	Not applicable
Population characteristics	Not applicable
Recruitment	Not applicable
Ethics oversight	Not applicable

Note that full information on the approval of the study protocol must also be provided in the manuscript.

Field-specific reporting

Please select the one below that is the best fit for your research. If you are not sure, read the appropriate sections before making your selection.

☐ Life sciences ☐ Behavioural & social sciences ☒ Ecological, evolutionary & environmental sciences

For a reference copy of the document with all sections, see nature.com/documents/nr-reporting-summary-flat.pdf

Ecological, evolutionary & environmental sciences study design

All studies must disclose on these points even when the disclosure is negative.

Study description	In this study, we integrate seismically measured subsurface fault zone processes, geodetically measured surface displacements, and modeling of strike-slip creeping fault to comprehensively characterize and understand multiscale fault zone deformation processes along the CSAF. We apply our approach to the CSAF in California.
Research sample	In this study, we integrate the location, time, magnitude, and focal mechanism of 75,164 M_l≥1.0 earthquakes, 145 M_l≥ 4.0 earthquakes, and 355 repeating earthquakes from 1980 to 2015 to place high-resolution constraints on the fault structure of the CSAF.<br><br>The InSAR surface slip-rate estimates were obtained from Jolivet et al., (2015)10. The repeating earthquake catalog was obtained from Waldhauser and Schaff (2021)28. The relocated earthquake catalog was obtained from Waldhauser and Schaff (2008)24. The earthquake phase information and seismic waveforms are taken from the Northern California Earthquake Data Center, Northern California Seismic Network (https://ncedc.org/ncsn/). The estimated earthquake focal mechanisms are available at Mendeley Data (https://data.mendeley.com/datasets/34szj3jm6k/1).
Sampling strategy	We choose all data available from 1980 to 2015 because the station network started from 1980
Data collection	*Describe the data collection procedure, including who recorded the data and how.*
Timing and spatial scale	We choose all data available from 1980 to 2015 because the station network started from 1980
Data exclusions	To focus on the interseismic period and disregard the co- and early postseismic deformation due to the 1989 M6.9 Loma Prieta and 2004 M_w6.0 Parkfield earthquakes, we exclude earthquakes within the first 3 years after these events when calculating the occurrence rates of repeating earthquakes and focal mechanism properties.
Reproducibility	All results are based on fixed existing data and data processing procedures.<br>All attempts to repeat the experiment were successful.
Randomization	In each group, we either choose all available data or randomly choose the same number of data points based on the purpose.
Blinding	We collected all available data. We used multiple datasets and have a single model to explain multiple different datasets. All data are not related to participant. Blinding was not relevant to my study

Did the study involve field work? ☐ Yes ☒ No

Reporting for specific materials, systems and methods

We require information from authors about some types of materials, experimental systems and methods used in many studies. Here, indicate whether each material, system or method listed is relevant to your study. If you are not sure if a list item applies to your research, read the appropriate section before selecting a response.

Materials & experimental systems

n/a	Involved in the study	
x		Antibodies
x		Eukaryotic cell lines
x		Palaeontology and archaeology
x		Animals and other organisms
x		Clinical data
		Dual use research of concern
		Plants

Methods

n/a	Involved in the study	
x		ChIP-seq
x		Flow cytometry
x		MRI-based neuroimaging

Dual use research of concern

Policy information about dual use research of concern

Hazards

Could the accidental, deliberate or reckless misuse of agents or technologies generated in the work, or the application of information presented in the manuscript, pose a threat to:

No	Yes	
x		Public health
x		National security
x		Crops and/or livestock
x		Ecosystems
x		Any other significant area

Experiments of concern

Does the work involve any of these experiments of concern:

No	Yes	
x		Demonstrate how to render a vaccine ineffective
x		Confer resistance to therapeutically useful antibiotics or antiviral agents
x		Enhance the virulence of a pathogen or render a nonpathogen virulent
x		Increase transmissibility of a pathogen
x		Alter the host range of a pathogen
x		Enable evasion of diagnostic/detection modalities
x		Enable the weaponization of a biological agent or toxin
x		Any other potentially harmful combination of experiments and agents

Plants

Seed stocks	Not applicable
Novel plant genotypes	Not applicable
Authentication	Not applicable
